# Psychological characteristics of the rural poor from self-assessment and external assessment perspectives and their association with socio-demographic factors

**DOI:** 10.3389/fpsyg.2025.1566612

**Published:** 2025-04-23

**Authors:** Wei Wei, Yan Li, Jinwei Zhu

**Affiliations:** ^1^School of Teacher Development, Shaanxi Normal University, Xi'an, Shaanxi, China; ^2^Faculty of Education, Shaanxi Xueqian Normal University, Xi'an, Shaanxi, China

**Keywords:** rural poor population, rural non-poor population, psychological traits, socio-demographic characteristics, factor analysis

## Abstract

**Objective:**

This study aims to explore the differences in psychological traits between rural poor individuals from self-assessment and external assessment perspectives, and to examine the correlation between the main socio-demographic factors of the poor population and their psychological traits. The ultimate goal is to provide a scientific basis for the formulation of effective poverty alleviation policies and to promote the development of poverty psychology and anti-poverty psychology.

**Methods:**

The study involved 1,943 poor individuals and 1,889 non-poor individuals from over 80 natural villages across eight provinces(regions) in central China (Shanxi, Henan, Hubei), northwestern China (Shaanxi, Xinjiang, Qinghai), and southwestern China (Guizhou and Yunnan). The psychological trait levels of the poor rural population were assessed using the “Rural poor Population Psychological Trait Assessment Questionnaire.”

**Results:**

Independent samples t-test showed that, from both self-assessment and external assessment perspectives, the poor population scored significantly higher on traits of retractability and stubbornness, while scoring significantly lower on the trait of grit compared to the non-poor population. The results of hierarchical linear regression indicated that socio-demographic factors such as age, health status, family size, and the poverty degree of the poor individuals contributed to 5.2% of the variance in the formation of psychological traits among the rural poor population.

**Conclusion:**

From both self-assessment and external assessment perspectives, significant differences were observed between the poor and non-poor populations in terms of their evaluations on traits of retractability, grit, and stubbornness. This indicates that there is a notable disparity between the poor individuals’ own perceptions and cognitions and the external evaluations they receive. The socio-demographic factors of rural poor individuals contribute only 5.2% to the variance in the formation of their psychological traits. This suggests that the deeper socio-cultural roots underlying the formation of psychological traits in the poor population await further exploration.

## 1 Introduction

Poverty represents a formidable challenge on a global scale in the 21st century (Gweshengwe et al., 2020). According to data from 2019, approximately 1.3 billion individuals are languishing in poverty across 101 nations worldwide (United Nations Development Programme and Oxford Poverty and Human Development Initiative, 2019). In order to tackle this pervasive issue, the 2030 Agenda for Sustainable Development has set forth an ambitious objective of eradicating all forms of poverty on a global basis (Koehler, 2017). The realization of this goal is contingent upon a multitude of factors. Nevertheless, undertaking a comprehensive analysis of the psychological traits of the poor population and the factors that influence them is undeniably one of the pivotal research domains.

### 1.1 The concept of poverty and psychological poverty

Despite the extensive history of poverty research, there remains its definition, with varying discipline characterized by an individual’s or household’s income falling below the threshold of living standards universally acknowledged by society. However, it was not until 1981 that the World Bank formulated a definition of poverty, describing it as the inability of certain population groups to access socially recognized and universally available opportunities for nutrition, housing, and participation, stemming from capability deficiencies (World Bank, 1981). This was subsequently refined to portray poverty as a multidimensional construct, encompassing facets such as restricted opportunities, power voids, inadequate security, malnutrition, and compromised health (World Bank, 2001). In academic circles, since the late 1990s, the comprehension of poverty’s essence has broadened from the narrow “income-expenditure” viewpoint to embrace multidimensional standpoints, including health, housing, education, and social protection (Alkire et al., 2017; Pasha, 2017; Alkire and Seth, 2015). This expanded understanding encompasses not merely deficiencies in food, nutrition, empowerment, education, health, transportation, and income, but also the erosion of human dignity, missed opportunities, and diminished satisfaction (Alkire and Foster, 2011; Arndt et al., 2016; Chen and Ravallion, 2008; Si et al., 2015; Dufford et al., 2019, 2020).

The term “psychological poverty” was initially introduced within the framework of the “culture of poverty” concept proposed by Lewis (1959). He posited that individuals who live in prolonged states of poverty develop distinct lifestyles, behavioral norms, and value systems, collectively forming a “poverty subculture.” The intergenerational transmission of this subculture further entrenched the state of poverty, resulting in psychological poverty. Later, Lewis (1966) characterized poverty as a unique subculture with its own set of norms and values. During the 1960s, the concept of psychological poverty was further developed within McClelland’s (1961) theory of achievement motivation. According to this theory, achievement motivation involves setting challenging yet attainable personal goals through individual effort, and it is an attribute that can be acquired through learning. Psychological poverty arises from a deficiency in achievement motivation. Some researchers define poverty as a condition characterized by low income and unmet basic needs (Mani et al., 2013), a subjective perception of one’s social status (Kraus et al., 2009, 2012; Kraus and Keltner, 2013), and as an indicator reflecting childhood socioeconomic status (Griskevicius et al., 2013; Mittal and Griskevicius, 2014). Consequently, contemporary perspectives on poverty have transcended the objective attributes of economic deprivation and increasingly regard it as a psychological trait, termed “psychological poverty.” American sociologist Inkeles (1985) posits that psychological poverty is characterized by a state in which an individual’s ideals, values, and mental states lag behind the dominant material modes of production in society. This manifests through certain outdated customs and behavioral patterns such as shortsightedness, inferiority complexes, negativity, withdrawal, conservatism, closed-mindedness, lack of willpower, and contentment with the status of poverty. Dalton et al. (2016) define psychological poverty as the “aspiration failures” and “behavioral failures” among the poor class resulting from prolonged material deprivation. These psychological and behavioral patterns are detrimental to escaping poverty. Aspiration failures refer to the absence of personal ambitions and distorted beliefs and awareness; behavioral failures denote a misalignment between capabilities and behavioral approaches, leading to decisions and actions that hinder poverty-alleviation.

Psychological poverty is a state of mind that is both associated with and relatively independent of an individual’s inadequate material living conditions. Individuals exhibiting traits of psychological poverty have psychological needs that are stalled at a lower level, showing an excessive preoccupation with the possession of material resources and property, while their aspirations for higher-level development are stymied. In other words, psychological poverty is an internal experience characterized by a cognitive inclination towards materialistic values and a behavioral proclivity for material possession, accompanied by negative emotional states such as insecurity and anxiety.

### 1.2 Psychological and behavioral characteristics of the poor population

From a psychological standpoint, poverty encompassequate economic earnings and a poverty encompasses not merely inadequate economic earnings and a scarcity of vital resources, but also encompasses disturbances in psychological functioning and deviations in behavior, among other psycho-behavioral difficulties. Researchers have highlighted that the poor populace displays distinct psychological and behavioral attributes that set them apart from other societal strata (Shah et al., 2012), with the majority of these traits being disadvantageous to their adaptation to contemporary society. These traits obstruct their personal growth and ascension in social stratification (Kraus et al., 2011). Furthermore, it is crucial to acknowledge that individuals residing in poverty constitute a heterogeneous collective (Ali-Akpajiak and Pyke, 2003). Admittedly, those with limited income not only grapple with inferior occupational status or a dearth of job prospects, which consequently diminishes their societal engagement, compromises their health, and even curtails life expectancy (Maxwell and Kenway, 2000), but they are also prone to elevated sentiments of helplessness, mortification, inferiority, and a weakened sense of self-worth. This, in turn, diminishes their perception of empowerment and command, while heightening their receptivity to social exclusion (Patel et al., 2000). Poverty is intertwined with elevated levels of stress, anxiety, despondency, and other adverse emotional states, potentially augmenting the likelihood of mood disorders (Ridley et al., 2020). A cross-national investigation by Walker et al. (2013) unveiled that a profound sense of shame pervades the poor across all surveyed nations, a characteristic that is consolidated through the dynamic interplay between the poor and their non-poor counterparts. This reinforcement fosters an augmented propensity for the poor to become estranged from mainstream societal norms and relegated to the peripheries (Walker et al., 2013; Gordon et al., 2000; Hobcraft and Kiernan, 2001), thereby exacerbating their psychological plight of destitution. Moreover, the detrimental stereotypical perceptions harbored against the poor can give rise to stigmatization, undermining their self-esteem and diluting their capacity for agency (Laurin et al., 2019).

Building upon the foundational principles of social comparison theory (Festinger, 1954), it is posited that an individual’s self-concept emerges from a relational calculus with others. Consequently, the socioeconomic status (SES) perception of poor individuals is constructed through comparative assessment against their non-poor counterparts (Kraus et al., 2009, 2012). Empirical evidence supports this notion, demonstrating that an individual’s relative income standing within a group exerts a more potent influence on life satisfaction than absolute income metrics (Boyce et al., 2010). Furthermore, subjective appraisals of SES have been shown to wield greater predictive power for well-being outcomes compared to objective SES indicators (Anderson et al., 2012), with a robust positive correlation established between income levels and subjective well-being (Haushofer and Salicath, 2023). Thus, social cognitive frameworks designate subjective SES as a pivotal structural determinant in gauging psychological poverty (Kraus et al., 2012). The culture of poverty thesis elucidates that a distinct cultural milieu pervasive among poor populations profoundly shapes behavioral propensities, value systems, and attitudes toward life (Lewis, 1998). In rural poor contexts, this culture materializes through derogatory attitudes towards education, a dearth of ambition, and a resignation to current circumstances. These cultural attributes not only serve to constrict the behavioral repertoire and psychological states of poor individuals but also erect formidable barriers to escaping the cycle of poverty. They also furnish a vital theoretical substratum for delving into the genesis of psychological traits observed within this demographic. Human capital theory propounds that such behavioral manifestations are symptomatic of an underlying deficiency in human capital, engendered by limited access to education, truncated work experience, and inadequate financial literacy (Lusardi and Mitchell, 2014). This multifaceted theoretical synthesis underscores the intricate interplay between social cognition, cultural context, and human capital in perpetuating and perpetuating psychological poverty.

The “just world” theory posits that an individual’s placement within the social hierarchy is a consequence of their deserving it. Poverty is frequently ascribed to personal characteristics, behavioral patterns, and decision-making frameworks. Lerner (1980) postulated a “just world” where individuals are bestowed with status and authority based on their psychological attributes, suggesting that the economically disadvantaged predominantly possess negative psychological traits. Empirical research has corroborated that individuals who staunchly adhere to the belief in a “just world” are more prone to harbor negative attitudes towards those in poverty (Cozzarelli et al., 2002; Furnham and Gunter, 1984). Investigations have demonstrated that, in comparison to groups with higher socioeconomic standing, the poor exhibit fewer positive attributes, such as intelligence, integrity, and capability (Lott, 2012; Mattan et al., 2017; Varnum, 2013). Neuroscientific inquiries have unveiled significant disparities in the neural processing mechanisms and cognitive performance of individuals originating from lower socioeconomic strata (Hackman et al., 2010; Noble et al., 2015). These findings illustrate that socioeconomic status not only influences an individual’s access to quality healthcare and educational opportunities but also exerts profound and consequential effects on brain functioning, cognitive growth, and educational accomplishments (Rakesh and Whittle, 2021).

A comprehensive review of the extant literature has unveiled two pivotal insights. Firstly, preceding studies have predominantly concentrated on the negative psychological attributes prevalent among rural poor demographics, encompassing traits such as diminished self-worth, anxiety disorders, and social reticence, whilst inadequately acknowledging their positive psychological characteristics. In actuality, rural inhabitants facing poverty also embody positive psychological traits, including resilience, industriousness, and optimism, which are instrumental in their endeavors to overcome poverty and attain prosperity. Neglecting the examination of these affirmative psychological features impedes a holistic comprehension of the psychological landscape of rural poor populations and may compromise the precision and efficacy of poverty alleviation strategies. Secondly, notwithstanding some research endeavors that have delved into the correlation between the psychological traits of rural poor populations and specific factors, there exists a paucity of rigorous theoretical dissection and empirical inquiry into the mechanisms through which these factors shape and influence the genesis and progression of psychological traits. For instance, the pathways by which socio-demographic variables—such as age, health status, educational level, and family size—affect the formation and evolution of psychological characteristics in rural poor populations remain inadequately elucidated and warrant further scholarly attention. With these considerations in mind, this study adopts a social psychological lens to scrutinize the disparities in psychological traits of rural poor individuals as perceived from their subjective standpoint versus external assessments. Additionally, it endeavors to probe the interconnections between salient socio-demographic determinants and their psychological profiles. This research aspires to furnish a robust scientific foundation for the crafting of efficacious poverty alleviation policies and to propel the scholarly disciplines of poverty psychology and anti-poverty psychology forward.

## 2 Methods and procedure

### 2.1 Participants

This research adopts a stratified random sampling approach, targeting eight provinces (regions) spanning Central China (Shanxi, Henan, Hubei), Northwest China (Shaanxi, Xinjiang, Qinghai), and Southwest China (Guizhou and Yunnan). Within these designated areas, over 80 natural villages were randomly selected, and 2,200 poor individuals aged 20 and above were surveyed. These participants had been verified and registered in the “National Poverty Alleviation Information System” by local township authorities. A total of 2,200 copies of the “Psychological Trait Assessment Questionnaire for Rural Poor Populations” were distributed for self-assessment. Out of these, 2,068 questionnaires were returned (yielding a response rate of 94%). After excluding incomplete or invalid questionnaires (totaling 125), 1,943 valid questionnaires were included in the statistical analysis, corresponding to an eligibility rate of 94%. The distribution of valid questionnaires across provinces (regions) was as follows: Shanxi (*n* = 195), Henan (*n* = 208), Hubei (*n* = 171), Shaanxi (*n* = 581), Xinjiang (*n* = 226), Qinghai (*n* = 198), Guizhou (*n* = 216), and Yunnan (*n* = 148). Among the respondents, there were 1,040 males (53.5%) and 903 females (46.5%). Furthermore, from the aforementioned eight provinces (regions), an additional random selection of over 80 natural villages was conducted, targeting 2,200 non-poor individuals aged 20 and above. The purpose was to evaluate the psychological traits of the poor population. Each participant was administered a copy of the “Psychological Trait Assessment Questionnaire for Rural Poor Populations,” resulting in the retrieval of 2,020 questionnaires (yielding a response rate of 91.8%). Following the exclusion of incomplete or invalid questionnaires (totaling 131), 1,889 valid questionnaires were retained for statistical analysis, corresponding to an eligibility rate of 93.5%. The distribution of these valid questionnaires across provinces (regions) was as follows: Shanxi (*n* = 292), Henan (*n* = 158), Hubei (*n* = 171), Shaanxi (*n* = 701), Xinjiang (*n* = 209), Qinghai (*n* = 139), Guizhou (*n* = 106), and Yunnan (*n* = 113). Among these respondents, there were 1,025 males (54.3%) and 864 females (45.7%).

### 2.2 Rural poor population psychological trait assessment questionnaire

This research adopts the “Psychological Trait Assessment Questionnaire for Rural Poor Populations” (Zhu et al., 2022) as its investigative tool. The questionnaire consists of 12 items, categorized into three subscales: retractability, grit, and stubbornness. The internal consistency reliability coefficients (Cronbach’s alpha) for the items within each subscale are 0.644, 0.612, and 0.599, respectively. The validity assessments yield satisfactory results (*χ^2^/df* = 2.710, *RMSEA* = 0.059, *SRMR* = 0.050, *CFI* = 0.850, *TLI* = 0.805). The correlation coefficients between all items and their corresponding factor scores exceed 0.61 (*p* < 0.001), indicating adequate item discriminant validity. Furthermore, the factor loadings of all items on their respective factors range from 0.66 to 0.73. Each item in the questionnaire pertains to descriptions of individual behavioral habits or personality traits. The rural poor participants were instructed to rate each item on a 5-point Likert scale (1 = strongly disagree; 2 = disagree; 3 = somewhat agree; 4 = agree; 5 = strongly agree) based on the extent to which the item aligns with their usual behavior or personality traits. Additionally, the rural non-poor individuals were asked to make judgments regarding the behavioral habits or personality traits of the poor population based on their observations, using the same 5-point scale.

The questionnaire incorporates socio-demographic variables pertinent to the respondents, encompassing age, gender, ethnicity, health status, educational level, family size, main occupation, source of economic income, poverty duration, intergenerational poverty, poverty degree, and familial influence, etc.

## 3 Results

### 3.1 Comparison of psychological trait assessments from self-assessment and external assessment perspectives among rural poor population

To examine the differences in psychological traits between how rural poor individuals perceive themselves and how non-poor individuals perceive those who are poor, an independent sample t-test was conducted based on survey data from both poor (*n* = 1943) and non-poor samples (*n* = 1889). The results are presented in Table 1.

**Table 1:** Differences in psychological trait assessments between rural poor population and non-poor population regarding the poor.

Factors	Poor population (*n* = 1943)		Non-poor population (*n* = 1889)		*t*
	*M*	SD	*M*	SD	
Retractability	2.698	0.796	2.342	0.830	13.511***
Grit	3.178	0.747	3.348	0.751	−7.057***
Stubbornness	2.713	0.770	2.468	0.789	9.710***

*p < 0.05; **p < 0.01; ***p < 0.001 (the same below).

From Table 1, it can be observed that on the characteristic of retractability, the poor population scored significantly higher (2.698 ± 0.796) than the non-poor population (2.342 ± 0.830), with a significant difference (*p* < 0.001). On the characteristic of grit, the poor population scored significantly lower (3.178 ± 0.747) than the non-poor population (3.348 ± 0.751), also with a significant difference (*p* < 0.001). On the characteristic of stubbornness, the poor population scored significantly higher (2.713 ± 0.770) than the non-poor population (2.468 ± 0.789), showing a significant difference (*p* < 0.001). It is evident that from both self-assessment and external assessment perspectives, the poor population significantly differs from the non-poor population in terms of retractability, grit, and stubbornness traits. This indicates that social environment and rural life experiences have profound and complex impacts on the formation of individual psychological traits. In light of this, this article further delves into the individual and socio-cultural roots behind the formation of these unique psychological traits among the rural poor population.

### 3.2 The predictive role of socio-demographic factors on the psychological traits of rural poor population

To examine the predictive role of socio-demographic factors on the psychological traits of the rural poor population, we constructed stratified regression models for each factor constituting the psychological traits of the poor population based on the results of correlation analysis from the sample (*n* = 1943). By identifying the incremental validity of these socio-demographic factors on each psychological trait factor, we explored the individual and socio-cultural foundations underlying the psychological traits of the rural poor population.

#### 3.2.1 Correlation analysis between socio-demographic factors and psychological traits of the rural poor populations

First, the data from sample (*n* = 1943) were used to calculate the Eta series correlation method between each dimension of poverty-alleviation behavioral strategies and the age, education level, health status, family size, poverty duration, poverty degree, main occupation, and income sources of the subjects, intergenerational poverty and other socio-demographic characteristics, and use Eta correlation analysis technology $F=\frac{E^{2}/\left(k-1\right)}{\left(1-E^{2}\right)/\left(n-k\right)}$ (In the formula: *E* = Eta correlation coefficient; *k* = number of variable categories; *n* = sample size) (Wen and Xing, 2001) tested the significance of the Eta coefficient, and the results (see Table 2) show: (1) Retractability exhibit significant correlations with age, health status, family size, poverty duration, main occupation, and income sources. (2) Grit demonstrate significant associations with age, family size, poverty degree, main occupation, and income sources. (3) Stubbornness are significantly related to health status, family size, poverty duration, and income sources. Overall, the psychological traits that constitute the characteristics of the poor population show relatively strong correlations with various socio-demographic factors of the poverty group and possess predictive validity. To further examine this, hierarchical regression analysis was utilized to construct cumulative models assessing the predictive role of socio-demographic factors on the psychological traits of the rural poor population.

**Table 2:** The correlation between socio-demographic factors and psychological traits of the poor population.

Social demographic characteristics		Retractability		Grit		Stubbornness	
		*Eta*	*F*	*Eta*	*F*	*Eta*	*F*
Individual characteristics	Age (*df* = 3,1939)	0.070	3.187*	0.067	2.921*	0.038	0.941
	Education level (*df* = 3,1939)	0.033	0702	0.047	1.418	0.056	2.009
	Health status (*df* = 2,1940)	0.073	5.225**	0.019	0.333	0.096	8.946***
Family characteristics	Family size (*df* = 2,1940)	0.068	4.573**	0.061	3.569*	0.073	5.204**
	Poverty duration (*df* = 3,1939)	0.076	3.773**	0.053	1.796	0.068	3.032*
	poverty degree (*df* = 2,1940)	0.037	1.350	0.061	3.566*	0.054	2.866
Social characteristics	Main occupation (*df* = 3,1939)	0.065	2.784*	0.068	2.975*	0.049	1.581
	Income sources (*df* = 2,1940)	0.088	7.646***	0.106	11.016***	0.090	7.932***
	Intergenerational poverty (*df* = 2,1940)	0.033	1.029	0.040	1.592	0.019	0.354

#### 3.2.2 The predictive role of social-demographic characteristics on retractability

Table 3 presents the results of hierarchical regression analysis on the impact of individual characteristics (age, health status, education level), family characteristics (family size, poverty duration, poverty degree), and social characteristics (intergenerational poverty, main occupation, income source) of poor individuals on their withdrawal-related gain validity. In the first step of the regression analysis, the regression coefficient for the predictor “age” was not significant (*t* = −2.427, *p* = 0.015), and the model’s coefficient of determination was also not significant (*F* = 5.892, *p* = 0.015), indicating that this factor could not effectively explain the dependent variable. The regression coefficient for the predictor “family size” was significant (*t* = −2.758, *p* = 0.006), and the model’s coefficient of determination was also significant (*F* = 7.607, *p* = 0.006), indicating that this factor could effectively explain the dependent variable. The regression coefficient for the predictor “intergenerational poverty” was not significant (*t* = 1.210, *p* = 0.227), and the model’s coefficient of determination was also not significant (*F* = 1.463, *p* = 0.227), indicating that this factor could not effectively explain the dependent variable. In the second step of the regression analysis, the regression coefficient for the newly added predictor “health status” was significant (*t* = 4.301, *p* < 0.001), and the newly added model’s coefficient of determination was also significant (*F* = 12.224, *p* < 0.001), indicating that the new factor could effectively explain the dependent variable. The regression coefficient for the newly added predictor “poverty duration” was not significant (*t* = −0.659, *p* = 0.510), and the newly added model’s coefficient of determination was also not significant (*F* = 4.019, *p* = 0.018), indicating that this factor could not effectively explain the dependent variable. The regression coefficient for the newly added predictor “main occupation” was not significant (*t* = 1.659, *p* = 0.097), and the newly added model’s coefficient of determination was also not significant (*F* = 2.109, *p* = 0.122), indicating that the new factor could not effectively explain the dependent variable. In the third step of the regression analysis, the regression coefficient for the newly added predictor “education level” was not significant (*t* = −1.206, *p* = 0.228), but the newly added model’s coefficient of determination was significant (*F* = 8.636, *p* < 0.001), indicating that the new factor could not effectively explain the dependent variable. The regression coefficient for the newly added predictor “poverty degree” was not significant (*t* = 0.662, *p* = 0.508), and the newly added model’s coefficient of determination was also not significant (*F* = 2.825, *p* = 0.037), indicating that this factor could not effectively explain the dependent variable. The regression coefficient for the newly added predictor “income source” was not significant (*t* = −0.517, *p* = 0.606), and the model’s coefficient of determination was also not significant (*F* = 1.494, *p* = 0.214), indicating that the new factor could not effectively explain the dependent variable. It is evident that the health status and family size factors of poor individuals can effectively explain the psychological traits related to retractability, with a combined explanatory rate of 1.6%. The regression equation models are as follows: Y = 2.698 + 0.102X (X = health status); Y = 2.876–0.076X (X = family size).

**Table 3:** Presents the results of the hierarchical regression analysis examining the impact of socio-demographic factors on retractability traits among poor individuals.

Predictor variable type	Model		Non-standardized coefficient		Standard coefficient Beta	*t*	*F*	*R* ^2^	Adjusted *R^2^*
			*B*	*SE*					
Individual characteristics	1	Constant	2.817	0.052		53.874***	5.892	0.003	0.003
		Age	−0.045	0.019	−0.055	−2.427			
	2	Constant	2.698	0.059		45.767***	12.224***	0.012	0.011
		Age	−0.074	0.020	−0.090	−3.754***			
		Health status	0.102	0.024	0.103	4.301***			
	3	Constant	2.788	0.095		29.232***	8.636***	0.013	0.012
		Age	−0.080	0.020	−0.098	−3.939***			
		Health status	0.096	0.024	0.097	3.927***			
		Education level	−0.024	0.020	−0.030	−1.206			
Family characteristics	1	Constant	2.876	0.067		42.911***	7.607**	0.004	0.003
		Family size	−0.076	0.028	−0.062	−2.758**			
	2	Constant	2.899	0.076		38.341***	4.019	0.004	0.003
		Family size	−0.075	0.028	−0.061	−2.690**			
		Poverty duration	−0.011	0.017	−0.015	−0.659			
	3	Constant	2.860	0.096		29.829***	2.825	0.004	0.003
		Family size	−0.075	0.028	−0.062	−2.712**			
		Poverty duration	−0.010	0.017	−0.013	−0.580			
		Poverty degree	0.017	0.026	0.015	0.662			
Social characteristics	1	Constant	2.637	0.053		49.520***	1.463	0.001	0.000
		Intergenerational poverty	0.028	0.023	0.027	1.210			
	2	Constant	2.581	0.063		40.906***	2.109	0.002	0.001
		Intergenerational poverty	0.028	0.023	0.028	1.216			
		Main occupation	0.028	0.017	0.038	1.659			
	3	Constant	2.596	0.070		37.256***	1.494	0.002	0.001
		Intergenerational poverty	0.028	0.023	0.027	1.201			
		Main occupation	0.031	0.018	0.043	1.729			
		Income sources	−0.011	0.021	−0.013	−0.517			

Based on the above analysis, it is evident that health status and family size are effective predictors of the formation of retractability psychological traits in the poor population.

#### 3.2.3 The predictive role of social-demographic characteristics on grit

Table 4 presents the results of hierarchical regression analysis on the impact of individual characteristics (age, health status, education level), family characteristics (family size, poverty duration, poverty degree), and social characteristics (intergenerational poverty, main occupation, income source) of the poor population on their grit. In the first step of the regression analysis, the regression coefficient of the predictor “age” was significant (*t* = 2.789, *p* = 0.005), and the model’s coefficient of determination was also significant (*F* = 7.777, *p* = 0.005), indicating that this factor effectively explains the dependent variable. The regression coefficient of the predictor “family size” was not significant (*t* = 1.223, *p* = 0.221), and neither was the model’s coefficient of determination (*F* = 1.496, *p* = 0.221), meaning this factor does not effectively explain the dependent variable. Similarly, the regression coefficient of the predictor “intergenerational poverty” was not significant (*t* = 1.232, *p* = 0.218), and the model’s coefficient of determination was also not significant (*F* = 1.519, *p* = 0.218), indicating this factor does not effectively explain the dependent variable. In the second step of the regression analysis, the regression coefficient of the newly added predictor “health status” was not significant (*t* = −1.626, *p* = 0.104), but the incremental model’s coefficient of determination was significant (*F* = 5.214, *p* = 0.006). However, the newly added factor did not provide an effective explanation for the dependent variable. The regression coefficient of the newly added predictor “poverty duration” was not significant (*t* = −0.224, *p* = 0.823), and neither was the coefficient of determination of the newly added model (*F* = 0.773, *p* = 0.462), indicating this factor does not effectively explain the dependent variable. The regression coefficient of the newly added predictor “main occupation” was also not significant (*t* = 0.819, *p* = 0.413), and the model’s coefficient of determination was not significant either (*F* = 1.095, *p* = 0.335), showing that the new factor does not effectively explain the dependent variable. In the third step of the regression analysis, the regression coefficient of the newly added predictor “education level” was not significant (*t* = 1.537, *p* = 0.125), but the coefficient of determination of the newly added model was significant (*F* = 4.266, *p* = 0.005). However, the newly added factor did not provide an effective explanation for the dependent variable. The regression coefficient of the newly added predictor “poverty degree” was not significant (*t* = −2.374, *p* = 0.018), and neither was the coefficient of determination of the newly added model (*F* = 2.396, *p* = 0.067), indicating this factor does not effectively explain the dependent variable. The regression coefficient of the newly added predictor “income source” was not significant (*t* = 0.252, *p* = 0.801), and the model’s coefficient of determination was also not significant (*F* = 0.751, *p* = 0.522), showing that the newly added factor does not effectively explain the dependent variable. It is evident that the age of the poor population can effectively explain the psychological trait of grit, with an explanatory rate of 0.4%. The regression equation model is: Y = 3.049 + 0.049X (X = age).

**Table 4:** Presents the results of the hierarchical regression analysis examining the impact of socio-demographic factors on grit traits among poor individuals.

Predictor variable type	Model		Non-standardized coefficient		Standard coefficient Beta	*t*	*F*	*R* ^2^	Adjusted *R^2^*
			*B*	*SE*					
Individual characteristics	1	Constant	3.049	0.049		62.186***	7.777**	0.004	0.003
		Age	0.049	0.017	0.063	2.789**			
	2	Constant	3.092	0.056		55.698***	5.214**	0.005	0.004
		Age	0.059	0.019	0.076	3.176**			
		Health status	−0.036	0.022	−0.039	−1.626			
	3	Constant	2.983	0.090		33.220***	4.266**	0.007	0.005
		Age	0.067	0.019	0.086	3.467***			
		health status	−0.029	0.023	−0.031	−1.245			
		Education level	0.029	0.019	0.038	1.537			
Family characteristics	1	Constant	3.103	0.063		49.277***	1.496	0.001	0.000
		Family size	0.032	0.026	0.028	1.223			
	2	Constant	3.111	0.071		43.779***	0.773	0.001	0.000
		Family size	0.032	0.026	0.028	1.238			
		Poverty duration	−0.004	0.016	−0.005	−0.224			
	3	Constant	3.242	0.090		36.030***	2.396	0.004	0.002
		Family size	0.035	0.026	0.030	1.323			
		Poverty duration	−0.008	0.016	−0.011	−0.488			
		Poverty degree	−0.057	0.024	−0.054	−2.374			
Social characteristics	1	Constant	3.120	0.050		62.436***	1.519	0.001	0.000
		Intergenerational poverty	0.027	0.022	0.028	1.232			
	2	Constant	3.094	0.059		52.231***	1.095	0.001	0.000
		Intergenerational poverty	0.027	0.022	0.028	1.235			
		Main occupation	0.013	0.016	0.019	0.819			
	3	Constant	3.087	0.065		47.181***	0.751	0.001	0.000
		Intergenerational poverty	0.027	0.022	0.028	1.242			
		Main occupation	0.011	0.017	0.016	0.656			
		Income sources	0.005	0.020	0.006	0.252			

Based on the above analysis, it is evident that age is a significant factor in predicting the formation of grit psychological traits among the poor population.

#### 3.2.4 The predictive role of social-demographic characteristics on stubbornness

Table 5 presents the hierarchical regression analysis results of the individual characteristics (age, health status, education level), family characteristics (family size, poverty duration, poverty degree), and social characteristics (intergenerational poverty, main occupation, income source) of the poor population on the validity of stubbornness enhancement. In the first step of the regression analysis, the regression coefficient of the predictor “age” was not significant (*t* = −0.605, *p* = 0.545), and the coefficient of determination of the model was also not significant (*F* = 0.366, *p* = 0.545), indicating that this factor could not effectively explain the dependent variable. The regression coefficient of the predictor “family size” was significant (*t* = −3.008, *p* = 0.003), and the coefficient of determination of the model was significant (*F* = 9.046, *p* = 0.003), with this factor providing an effective explanation of 0.5% for the dependent variable. The regression coefficient of the predictor “intergenerational poverty” was not significant (*t* = 0.664, *p* = 0.507), and the coefficient of determination of the model was also not significant (*F* = 0.441, *p* = 0.507), indicating that this factor could not effectively explain the dependent variable. In the second step of the regression analysis, the regression coefficient of the added predictor “health status” was significant (*t* = 3.921, *p* < 0.001), and the coefficient of determination of the added model was significant (*F* = 7.873, *p* < 0.001), with the added factor providing an effective explanation rate of 0.8% for the dependent variable. The regression coefficient of the added predictor “poverty duration” was not significant (*t* = 0.228, *p* = 0.819), but the coefficient of determination of the added model was significant (*F* = 4.547, *p* = 0.010), with this factor providing an effective explanation of 0.5% for the dependent variable. The regression coefficient of the added predictor “main occupation” was not significant (*t* = 0.328, *p* = 0.743), and the coefficient of determination of the added model was also not significant (*F* = 0.274, *p* = 0.760), indicating that the added factor could not effectively explain the dependent variable. In the third step of the regression analysis, the regression coefficient of the added predictor “education level” was not significant (*t* = −1.151, *p* = 0.250), but the coefficient of determination of the added model was significant (*F* = 5.691, *p* < 0.001), with the added factor providing an effective explanation rate of 0.9% for the dependent variable. The regression coefficient of the added predictor “poverty degree” was not significant (*t* = −0.210, *p* = 0.834), but the coefficient of determination of the added model was significant (*F* = 3.044, *p* = 0.028), with this factor providing an effective explanation of 0.5% for the dependent variable. The regression coefficient of the added predictor “income source” was not significant (*t* = 0.854, *p* = 0.393), and the coefficient of determination of the added model was also not significant (*F* = 0.426, *p* = 0.735), indicating that the added factor could not effectively explain the dependent variable. It can be seen that the health status and education level factors of the poor population can effectively explain the psychological traits of stubbornness, with an explanation rate of 1.7%. The regression equation model is: Y = 2.720 + 0.085×_1_–0.022×_2_ (X_1_ = health status, X_2_ = education level). The family size, poverty duration, and poverty degree factors of the poor population can effectively explain the psychological traits of stubbornness, with an explanation rate of 1.5%. The regression equation model is: Y = 2.905–0.080×_1_ + 0.004×_2_–0.005×_3_ (X_1_ = family size, X_2_ = poverty duration, X_3_ = poverty degree).

**Table 5:** Presents the results of the hierarchical regression analysis examining the impact of socio-demographic factors on stubbornness traits among poor individuals.

Predictor variable type	Model		Non-standardized coefficient		Standard coefficient Beta	*t*	*F*	*R* ^2^	Adjusted *R^2^*
			*B*	*SE*					
Individual characteristics	1	Constant	2.742	0.051		54.131***	0.366	0.000	0.000
		Age	−0.011	0.018	−0.014	−0.605			
	2	Constant	2.637	0.057		46.134***	7.873***	0.008	0.007
		Age	−0.036	0.019	−0.046	−1.903			
		Health status	0.091	0.023	0.094	3.921***			
	3	Constant	2.720	0.092		29.415***	5.691***	0.009	0.007
		Age	−0.042	0.020	−0.053	−2.136			
		Health status	0.085	0.024	0.088	3.568***			
		Education level	−0.022	0.019	−0.028	−1.151			
Family characteristics	1	Constant	2.901	0.065		44.761***	9.046**	0.005	0.004
		Family size	−0.080	0.027	−0.068	−3.008**			
	2	Constant	2.893	0.073		39.567***	4.547**	0.005	0.004
		Family size	−0.081	0.027	−0.069	−3.015**			
		Poverty duration	0.004	0.017	0.005	0.228			
	3	Constant	2.905	0.093		31.328***	3.044*	0.005	0.003
		Family size	−0.081	0.027	−0.068	−3.005**			
		Poverty duration	0.003	0.017	0.005	0.203			
		Poverty degree	−0.005	0.025	−0.005	−0.210			
Social characteristics	1	Constant	2.681	0.052		52.025***	0.441	0.000	0.000
		Intergenerational poverty	0.015	0.023	0.015	0.664			
	2	Constant	2.670	0.061		43.706***	0.274	0.000	−0.000
		Intergenerational poverty	0.015	0.023	0.015	0.665			
		main occupation	0.005	0.016	0.007	0.328			
	3	Constant	2.646	0.067		39.214***	0.426	0.001	−0.001
		Intergenerational poverty	0.016	0.023	0.016	0.689			
		Main occupation	−0.001	0.017	−0.001	−0.032			
		Income sources	0.018	0.021	0.021	0.854			

The above analysis results indicate that the health status, education level, family size, poverty duration, poverty degree of the poor population can significantly predict the formation of stubborn psychological traits in the poor. Those who have poorer health statuses, lower education levels, larger family sizes, longer durations of poverty, and higher degrees of poverty are more likely to develop psychological traits characterized by impulsive behavior, short-sighted aspirations, and stubbornness. However, the explanatory power of these factors is relatively weak.

## 4 Discussion

This research delves into the psychological attributes of rural poor individuals from both self-assessment and external assessment standpoints, with a focus on investigating the predictive efficacy of socio-demographic elements on their psychological traits. The findings reveal conspicuous disparities between the psychological characteristics rated by the poor themselves and those assessed by their non-poor counterparts. Specifically, rural poor populations manifest heightened levels of retractability and stubbornness, coupled with diminished levels of grit in self- evaluation; whereas external evaluations may yield notably divergent outcomes due to the influence of social cognition. Individuals exhibiting reclusive psychological tendencies are prone to blame others or external forces, shy away from responsibilities, display timidity and withdrawal, and even succumb to self -abandonment; those characterized by tenacious traits tend to possess strong willpower, lead frugal and industrious lives, dare to assume responsibilities, and strive for self-reliance; while those with stubbornness characteristics exhibit irrational behavior, have nebulous goals, feel inferior because of their poverty, and demonstrate rigid thinking patterns. Among these three prevalent factors, “retractability” stands out as the most quintessential psychological trait of the poor population, which could be one of the crucial reasons why they struggle to extricate themselves from poverty. Poverty psychology theory posits that the prolonged state of poverty may induce individuals to develop negative emotions, retractability, stubbornness, stress, and other adverse psychological attributes, thereby causing the poor to exhibit a lack of initiative and a deficiency in social responsibility due to poverty dependence (Haushofer and Fehr, 2014; Fu et al., 2020). Tenacity reflects the positive psychological quality of the poor, bearing significant positive implications for the sustainable development of their poverty alleviation endeavors (Zhu and Li, 2022). Rural poor populations frequently grapple with heightened living pressures and challenges, such as financial hardships and scarcity of educational resources, which may predispose them to adopt a reclusive mindset when confronted with adversity. Concurrently, rural poor individuals may develop a self-preservation mechanism as a result of prolonged marginalization, namely, the obstinate trait of adhering rigidly to their own views or practices. Moreover, due to the absence of external support and encouragement, the poor are more susceptible to falling into a vicious cycle of negative self-perception, believing that they are incapable of altering their circumstances, hence demonstrating lower levels of tenacity. The study further uncovers that, on the whole, socio-demographic factors exert a certain predictive influence on the formation of psychological traits among the poor population, albeit this influence is exceedingly feeble. This suggests that the formation of psychological traits in the poor population is the outcome of a confluence of multifaceted and intricate factors, and the socio-demographic variables at the phenomenological stratum are inadequate in elucidating the formation mechanisms of such stable psychological traits, necessitating an exploration of their deeper explanatory variables at a more microscopic level.

The constraints imposed by external conditions and the deficiency of social capital primarily account for the emergence of negative psychological traits among the rural poor population. On one hand, rural areas, characterized by scarce resource endowments, adverse natural environments, and geographically isolated locations, present formidable barriers to the eradication of poverty and the development of their inhabitants. These obstacles result in repeated failures in transcending the boundaries of underdevelopment, culminating in diminished self-confidence and the adoption of regressive and obstinate psychological traits (Fu et al., 2020). Such negative psychological attributes induce the poor to engage in negative self-assessments, disconnecting themselves from future aspirations of poverty alleviation and development. Consequently, they become disinclined to generate the psychological impetus necessary for seeking poverty eradication and development, descending into a state of despondency, complacency, and a dearth of agency (Yang and Yang and Lu, 2019). Social cognition theory posits that prolonged economic destitution leads the poor to recognize that their goal attainment is largely contingent upon external factors. Over time, they gradually develop a social cognitive orientation (Bellezza et al., 2017; Fouad and Fitzpatrick, 2009; Zunshine, 2017; Diemer and Ali, 2009). This social cognitive inclination gives rise to psychological and behavioral characteristics that markedly diverge from those of other groups in terms of social interaction and self-perception (Carroll et al., 2017; Özabacı, 2011). These characteristics often impede the adaptability of the poor to the socio-economic development environment. For instance, they attribute wealth disparities to uncontrollable factors such as birth background and fortune, believing that even with increased efforts, altering the status quo is arduous, thereby losing confidence, becoming complacent, and forfeiting the endogenous motivation to escape poverty (Damigos et al., 2021; He et al., 2022; Ismail et al., 2022). On the other hand, the rural poor possess lower levels of social capital and reside in a lower socio-economic stratum, rendering them more susceptible to poverty discrimination, social prejudices, and various physical and mental afflictions. Research has demonstrated that social capital fosters social welfare and economic advancement (Yushar et al., 2023). It holds key significance not only for stimulating family consumption growth but also for significantly enhancing the welfare level of economically disadvantaged families, thereby exerting a broad and profound influence on poverty eradication, socio-economic gap narrowing, and regional sustainable development (Hasbiah et al., 2024). Hence, to effectively address rural poverty, it is imperative to adopt a comprehensive and systematic strategic approach. This encompasses access to educational opportunities, provision of physical and mental health care services, and enhancement of infrastructure and other forms of social capital. Such measures enable the precise tailoring of poverty alleviation strategies to the actual needs of rural areas, substantially augmenting the likelihood of extricating themselves from poverty (Haeruddin et al., 2022).

The rural poor population commonly exhibits traits of pettiness, lacks market rationality, possesses inadequate social knowledge, shows low social participation, and is deficient in innovative and entrepreneurial spirit. Their cultural and lifestyle practices are backward, with an excessive dependence on government relief and support. These negative psychological attributes present significant challenges to the implementation of poverty alleviation strategies. Research indicates that the emotional state, social pressure, and other psychological characteristics of poor groups influence household economic behavior (Guiso and Paiella, 2008; Carvalho et al., 2016; Haq et al., 2021). The psychological traits of the poor significantly affect their behavioral objectives, preferences, and choices, constituting one of the primary reasons for their poverty (Liu, 2016). Therefore, strengthening the psychological modernization of rural poor populations to gradually develop modern psychological traits such as openness, rationality, confidence, and proactive change is a fundamental goal in the post-poverty alleviation era. Psychological modernization refers to the psychological and behavioral adaptations individuals make in response to societal modernization (Li and Xu, 2011). Strengthening national psychological construction is not a new proposition but was first proposed by Dr. Sun Yat-sen and has been practiced internationally. Dr. Sun believed that due to internal and external troubles, Chinese people were often short-sighted, narrow-minded, indifferent, disorganized, confused, timid, risk-averse, and reluctant to participate in national affairs, which significantly contributed to the country’s backwardness. The core purpose of psychological construction is to enlighten the people, change their old, narrow, and negative psychological states, cultivate a positive and optimistic healthy national mindset, and unite the hearts and strength of the people to promote social development and progress (Wang et al., 2019). In the early 20th century, the United States launched a rural small town construction integrating urban and rural areas; after World War II, Japan initiated a rural reform movement based on local self-reliance and future orientation; in the 1950s, the Netherlands carried out an intensive land consolidation movement; and in the 1970s, South Korea launched the New Village Movement emphasizing “diligence, self-help, and cooperation.” These rural construction movements aimed to stimulate the autonomy and initiative of rural poor populations, foster their psychological modernization, awaken their awareness of building a better homeland, and achieve rural prosperity. According to positive psychology, the self as an agent is an initiator of conscious behavior, a chooser of proactive strategies and defense mechanisms, capable not only of understanding, learning, communicating, and adapting to the environment but also of acting towards self-set goals (Carr, 2008). Therefore, in poverty alleviation practices, systematically constructing a social psychological service system using the principles and methods of positive psychology, enhancing positive goal recognition, improving the specific psychological traits of rural poor populations, cultivating positive personalities such as perseverance and self-efficacy, while reducing negative personalities like reticence and stubbornness, is of great practical significance for the poor to escape poverty.

This research adopts dual perspectives of self-evaluation and external evaluation to holistically grasp the psychological traits of the rural poor population. Self-evaluation reflects the subjective experiences and perceptions of the poor individuals themselves, whereas external evaluation offers an objective assessment from an outsider’s standpoint. The integration of these two approaches facilitates a profound understanding that poverty transcends mere material scarcity, encompassing instead a complex interplay of psychological and social dimensions. For instance, prolonged exposure to poverty may engender feelings of inferiority and anxiety among affected individuals, which subsequently influence their decision-making processes and developmental capacities, thereby impacting the trajectory of poverty alleviation. Through meticulous research, it becomes feasible to devise more targeted and all-encompassing support mechanisms, thereby circumventing the shortcomings associated with exclusive reliance on material assistance in poverty relief efforts. Furthermore, an in-depth exploration of the psychological characteristics of the rural poor population aids in identifying the psychological and social factors that perpetuate the intergenerational transmission of poverty. Consequently, in the practical realm of poverty alleviation, it is imperative to firmly establish the concept of “fostering ambition prior to providing livelihood support,” with a focus on nurturing positive psychological attributes within the poor, promoting the transition from traits of retraction and obstinacy to those of resilience and resolve, and cultivating a survival ethos characterized by “poverty without the loss of ambition.” Only through such measures can we fundamentally address the fatalistic and helpless attitudes typified by waiting, dependency, and demanding behaviors prevalent among those escaping poverty, eradicate and mitigate the phenomenon of relapse into poverty post-alleviation, sustain the longevity and efficacy of government poverty alleviation initiatives, establish a lasting mechanism for consolidating achievements in poverty alleviation, and lay a robust human resource foundation for the strategy of rural revitalization.

## 5 Conclusion, limitations, and prospects

### 5.1 Conclusion

This research takes the psychological and behavioral characteristics of the rural poor in China as the starting point and employs a questionnaire survey method. Through empirical data analysis, it explores the differences in the evaluation of psychological traits of rural poor individuals from both self-assessment and external-assessment perspectives. Furthermore, it examines the predictive effect of socio-demographic factors on the psychological traits of the poor. The main conclusions of the study are as follows: (1) From both self-assessment and external assessment viewpoints, there exist significant differences in the evaluation of the traits of retractability, grit, and stubbornness between the poor and non-poor groups. This indicates that the subjective feelings and cognitions of the poor individuals themselves notably differ from external evaluations. (2) Socio-demographic factors of rural poor people merely account for 5.2% of the explanatory power regarding the formation of their psychological traits. This implies that the deep social and cultural roots underlying the formation of psychological traits of the poor population necessitate further in-depth exploration.

### 5.2 Limitations

1. This study was confined to specific rural locales, precluding the inclusion of diverse rural typologies, thereby limiting the generalizability of its findings to a broader spectrum of rural poor populations.
2. The research exclusively examined the formation mechanism of psychological traits in rural poor populations from a socio-demographic standpoint, without delving into more granular micro-level or overarching macro-level perspectives.
3. Given the intricate interplay and reciprocal influences between psychological phenomena and social determinants, ascertaining a causal nexus between the psychological profiles of rural poor individuals and socio-demographic variables poses significant challenges.

### 5.3 Prospects

1. Future research should promote the deep integration of multidisciplinary theories, including psychology, sociology, economics, and other fields. For instance, by organically integrating poverty culture theory and social capital theory, etc., construct a more comprehensive and systematic theoretical framework from individual, family, and social dimensions to deeply explore the formation mechanism of psychological characteristics of rural poor populations.
2. Future research plans to conduct experimental designs and intervention studies. By setting up experimental and control groups and implementing psychological intervention measures, it can more scientifically verify the influence of socio - demographic factors on the psychological characteristics of rural poor populations and evaluate the effectiveness of different intervention strategies. For example, carrying out psychological counseling programs targeting rural poor populations and comparing the psychological changes before and after the intervention will provide a basis for formulating effective poverty alleviation policies.
3. Future research can classify and study rural poor populations based on different rural regions and cultural characteristics, and compare the similarities and differences in psychological traits between rural poor populations and non - poor populations, which will provide a reference for formulating poverty alleviation policies adapted to local conditions.

## Data Availability

The original contributions presented in the study are included in the article/supplementary material, further inquiries can be directed to the corresponding author.
